# *Tiliacora triandra *(Colebr.) Diels leaf extract enhances spatial learning and learning flexibility, and prevents dentate gyrus neuronal damage induced by cerebral ischemia/reperfusion injury in mice

**Published:** 2017

**Authors:** Wachiryah Thong-asa, Panas Tumkiratiwong, Vasakorn Bullangpoti, Kasem Kongnirundonsuk, Kanokwan Tilokskulchai

**Affiliations:** 1 *Physiology Division, Animal Toxicology and Physiology Specialty Research Unit (ATPRU), Department of Zoology, Faculty of Science, Kasetsart University, Bangkok, Thailand *; 2 *Animal Toxicology and Physiology Specialty Research Unit (ATPRU), Department of Zoology, Faculty of Science, Kasetsart University, Bangkok, Thailand*; 3 *Faculty of Science and Technology, Bansomdejchaopraya Rajabhat University, Hiranruchi, Dhonburi Bangkok, Thailand*; 4 *Neuroscience Unit, Department of Physiology, Faculty of Medicine Siriraj, Siriraj Hospital, Mahidol University, Bangkok, Thailand*

**Keywords:** Spatial learning, Learning flexibility, Cerebral ischemia/reperfusion injury, Tiliacora triandra, Dorsal hippocampus

## Abstract

**Objective:**

The present study investigated the effects of a local Thai vegetable*, Tiliacora triandra* (Colebr.) Diels, also known as Yanang, against cerebral ischemia/reperfusion injury in mice.

**Materials and Methods:**

Thirty male ICR mice were divided into three experimental groups of BLCCAO + 10% Tween 80, BLCCAO + *T. triandra* 300 mg/kg, and BLCCAO + *T. triandra* 600 mg/kg. Cerebral ischemia/reperfusion was induced by three minutes of bilateral common carotid artery occlusion (BLCCAO) followed by 18 days of reperfusion. Leaf extract was administered orally 24 hours after arterial occlusion and continued for 18 consecutive days. Cognitive abilities were evaluated using the Morris water maze. Histological analysis was conducted in the dorsal hippocampus subregions CA1, CA3, and DG and white matter regions (the corpus callosum, internal capsule, and optic tract) using 0.1 % cresyl violet and 0.1% Luxol fast blue staining.

**Results:**

Results showed that *T. triandra *leaf extract at the doses of 300 and 600 mg/kg significantly enhanced spatial learning, and learning flexibility, and prevented neuronal death in the DG of mice following ischemia/reperfusion.

**Conclusion:**

*T. triandra* leaf extract enhanced spatial learning, and learning flexibility, and prevented DG neuronal death in a mice model of cerebral ischemia/reperfusion.

## Introduction

*Tiliacora triandra* (Colebr.) Diels, or Yanang, is a local Thai vegetable particularly used in northeastern cuisine. This climbing plant is known as a rejuvenating and neurotonic agent in traditional Thai remedies. Its anti-bacterial, anti-malarial, anti-cancer, anti-inflammation, anti-oxidant, anti-fever, alcohol detoxification, and acetylcholine esterase (AChE) inhibitory properties have been witnessed. Leaf extract is composed of condensed tannin, triterpene, flavonoid, saponin, phyrol and α-tocopherol. Polyphenols include p-hydroxybenzoic acid, minecoside, flavones glycoside cinnamic acids derivative, and monoepoxy-betacarotene (Phunchango et al., 2015 ▶; Kaewpiboon et al., 2014 ▶; Singthong et al., 2014 ▶; Sureram et al., 2012 ▶; Boonsong et al., 2009 ▶). Focusing on the anti-oxidant and anti-inflammatory properties of *T. triandra* leaf extract, therefore, might be beneficial in fighting against diseases involving free radicals and inflammation. 

Stroke-induced ischemic brain damage results in a variety of disabilities. Multiple cell death mechanisms, such as excitotoxicity, free radical formation, inflammation, and apoptosis, have been elucidated. The loss of oxygen and energy supply during an ischemia period in addition to reperfusion injuries during recirculation, cause delayed neuronal damage as a result of free radical formation and increased pro-inflammatory cytokines (Phillis et al., 2002 ▶; White et al., 2000 ▶). The presence of pro-inflammatory cytokines such as tumor necrosis factor (TNF) - alpha, interleukin (IL) - 1beta, and IL-6, from 3 to 24 hr. after transient global ischemia is a result of glial activation. The reactive species like oxygen and nitrogen radicals acting as potential oxidizing agents, damage cellular membrane through lipid peroxidation (LPO), and weaken the intrinsic anti-oxidant mechanisms such as superoxide dismutase (SOD), catalase (CAT), and glutathione peroxidase (GSH-Px) during an ischemia/reperfusion injury (Hou et al., 2002 ▶; White et al., 2000 ▶). Neuronal damage in susceptible brain areas such as the hippocampus, the basal ganglia, and the cerebral cortex leads to cognitive decline due to formation of reactive species and activation of pro-inflammatory cytokines. Deficits of cholinergic functions in particular brain areas are also observed (Hou et al., 2002 ▶). The anti-oxidant, anti-inflammatory, and AChE inhibitory properties of *T. triandra*, ameliorate the deteriorating effects of ischemia/reperfusion injury. Therefore, the present study investigated the effects of *T. triandra* leaf extract on cognitive abilities and neuronal damage in a mice model of cerebral ischemia/reperfusion. 

## Material and Methods

**Plant collection and extraction**

*T. triandra *leaves were collected from Ladyao, Jatujak (District), Bangkok, Thailand. The plant identity was confirmed by a plant taxonomist of ATPSRU, Faculty of Science, Kasetsart University. Air-dried *T. triandra* leaves were powdered and subsequently extracted with 95% ethanol (EtOH) using a Soxhlet extractor for 18 - 20 hr. Leaf extract was filtered and concentrated in a rotary vacuum. This process was repeated three times to obtain a dark green extract which was stored in an air-tight bottle at 4 °C until used.

**High performance liquid chromatography (HPLC) basic screening**

HPLC basic screening of *T. triandra* leaf extract was performed using Silica gel 60 (70-230 mesh ASTM, Merck, Germany) as the stationary phase and 50% methanol (MeOH) in water as the mobile phase. The flow rate was 0.3 ml/min and injection volume was 1 µl. The absorbance was read both in UV and visible wavelengths using an HPLC apparatus (Agilent Technologies, USA).

**Total phenolic determination**

Leaf extract of *T. triandra* (20 µl) was pipetted and mixed with 1.5 ml of distilled water and 100 µl of Folin-Ciocalteu reagent (Merck, Germany). After five minutes, 300 µl of 20% sodium carbonate solution was added (Merck, Germany), and the solution was shaken. The solution was kept at 40 ^°^C for 30 min before the absorbance was read at 765 nm (Biotek PowerWave 340, Canada). Three replicates of samples were used. Total phenolic content calculations were made using Gallic acid (Merck, Germany) standard curve (0, 2.5, 5.0, 7.5, 10, 20, and 30 mg/ml), and the total phenolic content was represented as mg Gallic acid equivalent (GAE)/g. 

**Total flavonoid determination**

The aluminum chloride colorimetric method was used to determine the total flavonoid content. Leaf extract (0.5 ml) was pipetted (100 mg/ml of 95% EtOH) and mixed with 1.5 ml of 95% EtOH, 0.1 ml of 10% aluminum chloride, 0.1 ml of 1M potassium acetate (Merck, Germany), and 2.8 ml distilled water. The mixture was incubated at room temperature for 30 min and read at 415 nm (Biotek PowerWaveXS, Canada). The total flavonoid content was calculated using Quercetin (Merck, Germany) standard curve (0, 25, 50, and 100 mg/ml) and represented as mg Quercetin equivalent (QE)/g.

**Animals**

Thirty male ICR mice (*Mus musculus*) weighing 40–50 g were obtained from the National Laboratory Animal Centre, Mahidol University, Salaya, Nakornprathom province and used in this study. Mice were housed under a 12h/12h light-dark cycle with well-controlled temperature (23 ± 2 ^o^C) and humidity (55 ± 5%) and animals had free access to standard food pellets and filtered water. This research was conducted in accordance with internationally accepted principles for laboratory animal use and care of the European Community (EEC directive of 1986; 86/609/EEC). The experimental protocol was approved by the Animal Ethics Committee, Kasetsart University Research and Development Institute (KURDI), Kasetsart University, Bangkok, Thailand (ID#OACKU 00158).

**Experimental protocol**

**Figure 1:**
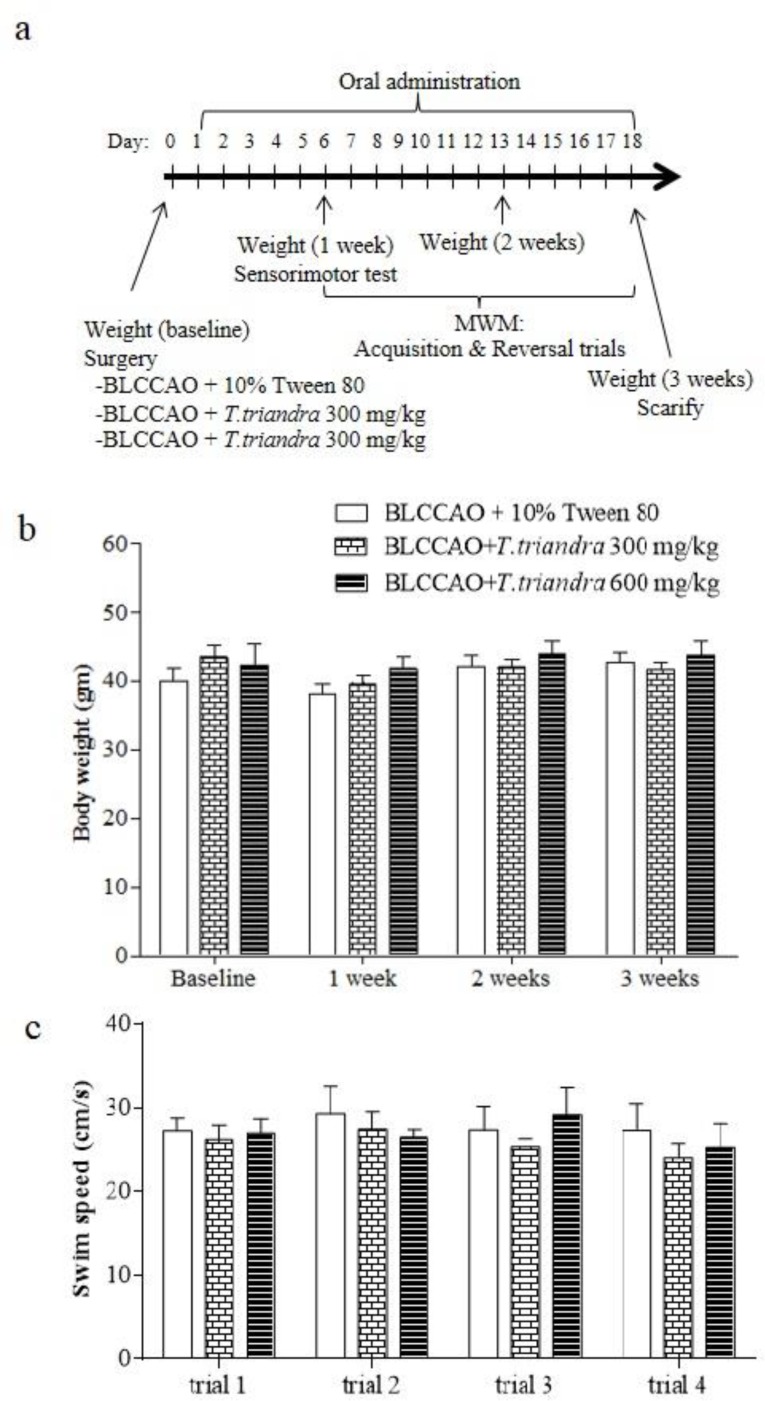
(a) Experimental protocol; (b) mice body weight at baseline, one, two, and three weeks after surgery; and (c) sensorimotor evaluation represented as swim speed in each trial of cue test

Mice were randomly assigned into three groups: BLCCAO + 10% Tween 80, BLCCAO + *T. triandra* 300 mg/kg, and BLCCAO + *T. triandra* 600 mg/kg (n = 10 for each group). Then, cerebral ischemia/reperfusion injury was induced using three minutes of bilateral common carotid artery occlusion. This ischemia/reperfusion model was chosen based on the instructions of Murakami et al. (1998) ▶ who used the same mouse strain and revealed a low mortality rate with sufficient ischemic effects on neuronal death in the vulnerable brain areas. After fasting, mice were anesthetized by sodium pentobarbital (45 mg/kg) intraperitoneal injection. After checking their reflexes, an incision was made in the skin on the midline ventral neck. Both common carotid arteries were exposed and cleared from nerves and surrounding connective tissues and then, transiently occluded by clipping. Clips were removed after three minutes to restore blood flow (reperfusion). After surgery, all wounds were sutured using silk sutures, and antibiotics were given through intramuscular injections before the mice were placed under heat lamps and blankets in the recovery box. The treatments were orally administered 24 hr. after surgery via gavage and continued for 18 days. The vehicle was 10% Tween 80. The treatments of 300 and 600 mg/kg of *T. triandra* leaf extract were prepared from a stock concentration of 300 mg/ml of *T. triandra* leaf extract in 10% Tween 80 (Figure 1a).

**Cognitive tests in the morris water maze**

The Morris water maze (MWM) was a plastic pool (150 cm in diameter and 50 cm tall), filled with tap water (25 ^°^C and 40 cm deep). Cognitive abilities of the mice were tested on day 7 after surgery (Figure 1a). Prior to cognitive testing (on day 6), a sensorimotor evaluation was performed to assess visual and motor abilities. Sensorimotor tests were conducted using the visible platform paradigm. Briefly, a visible platform was placed above the water surface (2 cm) for being easily seen by the mice. All mice were given four trials to swim, search, and sit on the visible platform. The maximum time for each trial was 120 sec. The swimming speeds of the mice in each group were recorded for sensorimotor evaluation. On the following day, spatial learning was tested; testing continued for five days as the acquisition trial (days 7-11). Briefly, the pool was divided into four quadrants, northeast (NE), northwest (NW), southeast (SE), and southwest (SW), by a cross mark set on the computer monitor that was connected to a ceiling camera. The hidden platform was submerged 2 cm under the water surface in the center of the NE quadrant (the target quadrant of the acquisition trial). A variety of visual cues were placed around the pool. Mice were continually given four trials a day for a maximum time of 120 sec in each trial. In out-of-time cases, the mice were guided to the hidden platform by the experimenter. In such cases, escape latency was recorded for a maximum time of 120 sec. When the acquisition trial was completed, the probe trial was conducted in order to determine memory capacity (day 12). The hidden platform was removed from the target quadrant and mice were allowed to swim for 60 sec. The time spent in each quadrant was recorded and converted to percentage of time spent in each quadrant to evaluate the memory capacity. On the following day, learning flexibility was assessed in the reversal trial (days 13-17). The only difference from the acquisition trial, was that the hidden platform was switched to the opposite quadrant (SW). The probe trial was also conducted on the last test day (day 18) to evaluate memory capacity for the new platform location. 

**Histological analysis**

 On day 18, all mice were sacrificed by an overdose intraperitoneal injection of sodium pentobarbital (> 60 mg/kg). Intracardiac perfusion using 0.9% normal saline solution (NSS) followed by 4% paraformaldehyde (PFA) in 0.1 M phosphate buffer saline (PBS) (pH 7.4), was induced. Brains were removed and stored in 4% PFA in 0.1M PBS (pH 7.4) for 24 hr. before they were processed and embedded in paraffin. Brain sections (5 µm thickness) of the dorsal hippocampus at bregma – 1.98 were collected (Paxinos and Franklin, 2008 ▶) and stained with 0.1% Luxol fast blue and 0.1% cresyl violet (Merck, Germany). Briefly, after deparaffinization and rehydration, brain sections were soaked in 0.1% Luxol fast blue and kept overnight in a hot air oven at 56 ^°^C. On the following day, brain sections were washed in 95% EtOH and differentiated in 0.5% lithium carbonate and 70% EtOH. Afterwards, brain sections were dipped in distilled water and stained with 0.1% cresyl violet for 30 sec, dehydrated using 95-100% EtOH and xylene, and mounted with a cover slide.

Brain photomicrographs were captured using Olympus Tg300 microscopy. Viable and dead neurons were counted in three captured images (613.26 × 478.29 µm of each image area) at 200x magnification. The areas of interest were the cornus ammonis (CA) 1, 3, and dentate gyrus (DG) of the dorsal hippocampus. A viable neuron was characterized by a visible nucleus and nucleolus with a light purple stain in the cytoplasm. The diameter of cells ranged between 15 and 35 µm in the CA1 and CA3 regions and 9 and 25 µm in the DG region. Dead neurons were characterized by dark purple staining of cresyl violet with cell shrinkage; the nucleolus was not noticeable, and a vacuole appeared around the cell. Viable and dead neurons were counted in a blind fashion by two investigators using the UTHSCSA Image Tool 3.0. Five brain sections were collected from each animal (space interval = 125 µm). The average of data was calculated and interpreted as percentage of dead cells per area using the following formula: 

% of dead cells = [dead cells /(viable cells + dead cells)] × 100 (Thong-asa and Tilokskulchai, 2014 ▶).

White matter areas were examined by the blue stained areas of Luxol fast blue. White matter density was interpreted as percentage of area of white matter in the areas of the corpus callosum, the internal capsule, and the optic tract using NIH Image J. Briefly, three images of white matter at 200x magnification were taken, avoiding the capillary or non-white matter parts which were converted to a binary image (black and white) so that the non-white matter stain and the threshold adjustment could be manually edited. Analysis of white matter density was represented as percentage of area.

**Statistical analysis**

Data was expressed as mean ± standard error of mean (SEM). Escape latencies (determination of spatial learning and learning flexibility) were analyzed by repeated-measure ANOVA followed by Fisher’s *post-hoc* test. Percentages of time spent in the target quadrant (determination of spatial memory), dead cells, and area of white matter were analyzed by one-way ANOVA followed by Fisher’s *post-hoc* test. Statistical significance was accepted as p<0.05.

## Results

***T. triandra*** **leaf extract**

The weight of dried *T. triandra* leaf powder was 2,000 g, which yielded 329.3 g after extraction. This was equivalent to 16.46% leaf extract yield. The HPLC chromatograms of *T. triandra* leaf extract revealed absorbance mainly within the UV wavelength. Several peaks were found at 205, 250, 280, and 300 nm. The HPLC results suggested that *T. triandra* leaf extract is mainly composed of polyphenolic compounds as revealed by the absorbance within the UV wavelength. The total phenol content calculated using a standard curve of Gallic acid (y = 0.0631x + 0.078, R^2^ = 0.99), was 340.21 ± 5.76 mg GAE/g. The total flavonoid content calculated using a standard curve of Quercetin (y = 0.0536x + 0.1786, R^2^ = 0.9973), was 231.29 ± 1.72 mg QE/g. 

**Animal health and sensorimotor evaluation**

The mortality rate was zero, and the body weight of all mice slightly increased during the three weeks of experimentation, but not significantly as shown in Figure 1b. This suggests that the ischemia/reperfusion procedure and the oral administration of *T. triandra *leaf extract for 18 consecutive days had no effect on the mice health. Sensorimotor evaluation represented by swim speed also showed no difference as shown in Figure 1c. After surgery and treatments, all mice displayed the same swimming and searching abilities for the visible platform. The results suggested that in mice with ischemia/reperfusion injury induced by a 3-min bilateral common carotid artery occlusion, the subsequent oral administration of 10% Tween 80 or *T. triandra* leaf extract, did not affect the mice health, mortality, or sensorimotor skills.

**Spatial cognition and learning flexibility**

**Figure 2.**
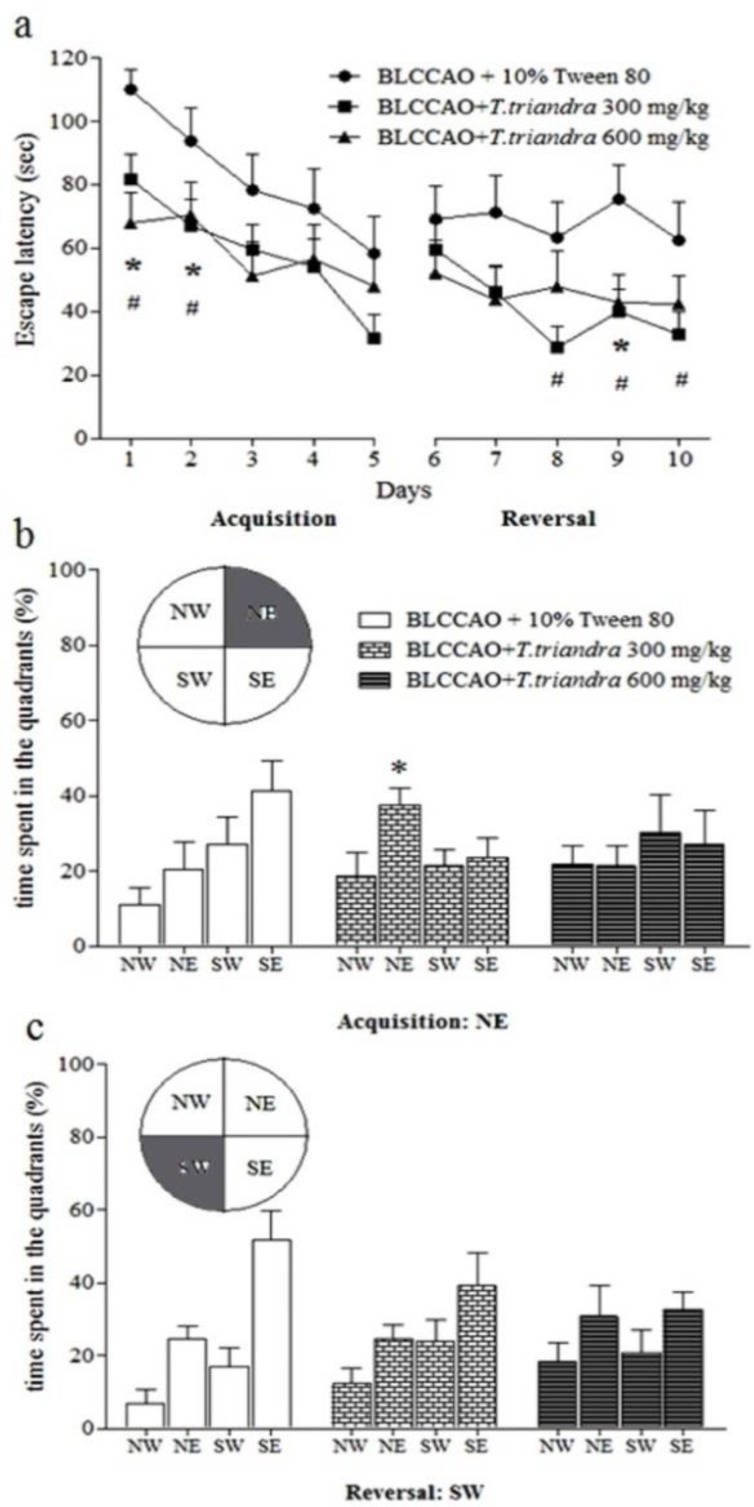
a) Spatial learning and learning flexibility represented by escape latencies; (b) spatial memory of acquisition probe trial; (c) spatial memory of reversal probe trial. * p<0.05 comparing BLCCAO + *T. triandra* 300 mg/kg with BLCCAO + 10% Tween 80; # p< 0.05 comparing BLCCAO + *T. triandra *600 mg/kg with BLCCAO + 10% Tween 80

*T. triandra* leaf extract enhanced spatial learning and learning flexibility in the animals treated with BLCCAO + *T. triandra*. Escape latency in acquisition and reversal trials of the BLCCAO + *T. triandra* 300 mg/kg and BLCCAO + *T. triandra* 600 mg/kg groups were significantly shorter than those of the BLCCAO + 10% Tween 80 group (p<0.05, Figure 2a). Significant enhancement of spatial memory was seen only in the BLCCAO + *T. triandra* extract 300 mg/kg group represented by more time spent in the target quadrant than the BLCCAO+10% Tween 80 mice (p<0.05, Figure 2b). The BLCCAO+*T. triandra* 600 mg/kg group spent more time in the target quadrant than the BLCCAO + 10% Tween 80 mice, but the difference was not significant. The BLCCAO groups showed a similar tendency for memory capacity to locate the new platform location in reversal probe trials (Figure 2c). The mice spent more time in the previous target quadrant and the adjacent quadrant, but less time in the new target quadrant. The results suggested that both 300 and 600 mg/kg doses of* T. triandra* leaf extract enhanced spatial learning and learning flexibility. However, only the dose of 300 mg/kg of *T. triandra* leaf extract enhanced spatial memory. 

**Histological analysis**

**Figure 3:**
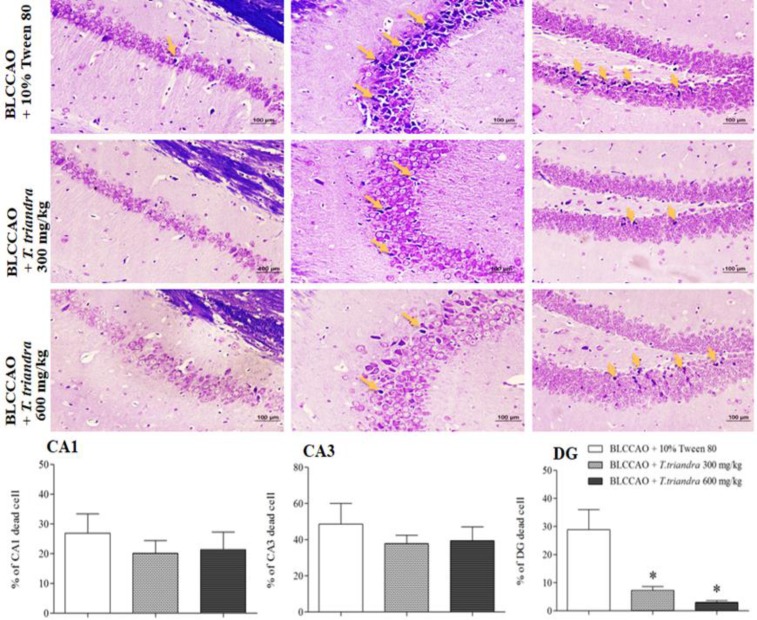
Photomicrographs at 200x magnification and histograms of the percentages of dead cells in the CA1, CA3, and DG areas of the dorsal hippocampus; arrows indicate dead cells, shrinkage and appearance of vacuole around cell with dark purple stain from 0.1% cresyl violet, * p<0.05 compared to BLCCAO + 10% Tween 80 (scale bar = 100 µm

 The percentages of dead cells in the CA1 and CA3 areas of the mice treated with BLCCAO + *T. triandra* 300 mg/kg and 600 mg/kg were lower than those of the mice treated with BLCCAO + 10% Tween 80, but it was not significantly different. Unlike in the DG area, a significant difference was found in the BLCCAO + *T. triandra* 300 mg/kg and 600 mg/kg groups when compared with the BLCCAO + 10% Tween 80 groups (p<0.05, Figure 3). The results suggested that *T. triandra* leaf extract given during the reperfusion period can prevent increases in neuronal death in the DG region of the dorsal hippocampus.

**Figure 4:**
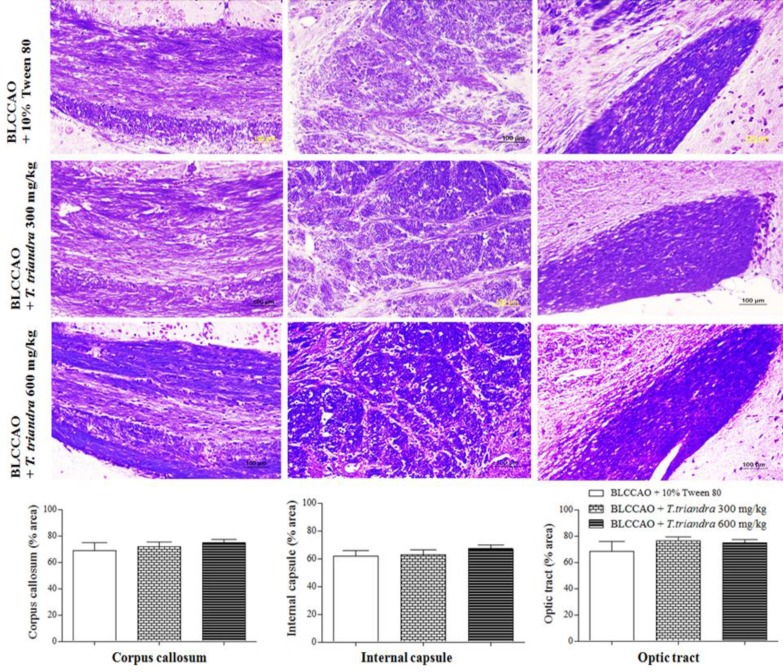
Photomicrographs of the corpus callosum, the internal capsule, and the optic tract at 200x magnification (scale bar = 100 µm). The histograms show the percentage (%) area of white matter density

White matter density did not differ in the corpus callosum, the internal capsule, and the optic tract. Nevertheless, the white matter density of mice in the BLCCAO + *T. triandra* 300 mg/kg and 600 mg/kg groups, was higher than that of the BLCCAO + 10% Tween 80 group (Figure 4).

## Discussion

 The present study elucidated the effects of *T. triandra *leaf extract on spatial learning, learning flexibility, and hippocampal neuronal death in mice with cerebral ischemia/reperfusion injury induced by three minutes of bilateral common carotid artery occlusion. The results showed that *T. triandra* at the doses of 300 and 600 mg/kg enhanced spatial learning and learning flexibility. The enhancement of spatial memory was found only at the dose of 300 mg/kg of* T. triandra*. The percentages of dead cells in the CA1, CA3, and DG regions of the BLCCAO + 10% Tween 80 mice were higher than in the *T. triandra*-treated groups. However, a significant difference was found only in the DG region. White matter density in the corpus callosum, the internal capsule, and the optic tract showed no difference. The results of the present study suggested that *T. triandra* leaf extract enhanced spatial learning, learning flexibility, and prevented neuronal death in the hippocampal DG region of mice with cerebral ischemia/reperfusion injury. The beneficial effects of *T. triandra* were related to high levels of total phenolic and total flavonoid contents of the leaf extract.The hippocampal cells responsible for spatial cognition are located in the CA1 region (e.g., place cells) and function together with the hippocampal network, the parahippocampal region, and the cerebral cortex (Broadbent et al., 2004 ▶). Significant differences in the percentages of dead cells in the CA1 and CA3 regions were not observed in the present study; however, the percentages of dead cells in the *T. triandra*-treated groups were slightly lower than those of the non-treated group and might involve enhancement of learning ability. Minimal damage to these vulnerable regions was induced by a 3-min bilateral common carotid artery occlusion as the ischemia/reperfusion model as reported by Murakami et al. (1998 ▶), using a short period of brain ischemia to cause sufficient hippocampal neuronal damage in a delayed fashion. As the reperfusion period was as long as 18 days, the protective effect of *T. triandra* leaf extract was obvious in the DG region. Its neuroprotective properties in the CA1 and CA3 regions together with significant protective effect in the DG region, are clearly related to the high phenolic content of *T. triandra *leaf extract. The phenolic compounds can prevent and reduce the activation of death mechanisms caused by excitotoxicity, free radical formation, inflammation, and apoptosis (Parihar and Hemnani, 2003 ▶; Hou et al., 2002 ▶). *T. triandra* had high phenolic contents with many medicinal properties such as anti-oxidant, anti-inflammation and AChE inhibitory properties that could counteract the activation of mechanisms of death and enhance cognitive abilities (Phunchango et al., 2015 ▶; Kaewpiboon et al., 2014 ▶; Singthong et al., 2014 ▶; Sureram et al., 2012 ▶; Boonsong et al., 2009 ▶). The phytochemicals that were found in *T. triandra* leaf extract, i.e. polyphenols, flavonoids, saponins, phyrol, and α-tocopherol, are well-known anti-oxidant and anti-inflammatory agents. High total phenolic and flavonoid contents of *T. triandra* leaf extract were determined in the present study. Moreover, recent evidence has proven that polyphenols exhibit neuroprotective potential, and memory, learning, and cognition-promoting effects (Phunchango et al., 2015 ▶). The present study clearly revealed that there is no dose-dependency in the enhancement of spatial learning ability, learning flexibility, or spatial memory capacity. *T. triandra* leaf extract in both 300 mg/kg and 600 mg/kg doses significantly enhanced spatial learning ability and learning flexibility; however, spatial memory capacity was not enhanced in the group treated with *T. triandra* 600 mg/kg. Different circuits of memory acquisition, consolidation, and retrieval were all involved in the activation of NMDA receptors, but in unique pathways (Logue and Gould, 2014 ▶; Ragozzino et al., 2002 ▶). The high dose (600 mg/kg) of *T. triandra* leaf extract had no effect on spatial memory (acquisition probe). Moreover, neither the 300 mg/kg dose nor the 600 mg/kg dose affected spatial memory of the new platform location (reversal probe). Analysis of polyphenol in *T. triandra* leaf extract identified p-hydroxybenzoic acid, minecoside, flavones glycoside cinnamic acids derivative, and monoepoxy-betacarotene (Singthong et al., 2014 ▶; Boonsong et al., 2009 ▶). It has been reported that p-hydroxybenzoic acid exhibits sedative and hypnotic effects in mice. This might influence motivation in spatial memory tests as shown in the present study. Evidence shows that most polyphenols act in a bell-shaped dose-response manner, presenting cellular toxicity at high concentrations, but they have cellular benefits at low concentrations (Farhoosh et al., 2016 ▶; Khan et al., 2016 ▶). A report of sub-chronic oral toxicity testing revealed that a 90-day treatment with *T. triandra* leaf extract at 300, 600, and 1,500 mg/kg doses induces no sign of toxicity; however, the psychological effects of *T. triandra* leaf extract might be seen (Sireeratawong et al., 2008 ▶). 

The present study showed dose-dependent responses only for percentage of dead cells in DG; 600 mg/kg of *T. triandra* leaf extract represented the lowest percentage of dead cell. Conclusions regarding all pathways cannot be reached with only one brain region examination. Rather than the protective effect of *T. triandra* leaf extract on DG neurons, the activation of neurogenesis is proposed. Two major neurogenic regions in the adult mammalian central nervous system are the sub-ventricular zone and the sub-granular layer of the DG region. Newly-born cells from the sub-granular layer can become granule cells of the DG region, and they can be increased by neurogenesis under many conditions such as environmental enrichment, exercise, learning and memory tasks, epilepsy, stroke, traumatic brain injury, and Huntington’s and Alzheimer’s diseases (Luikart et al., 2012 ▶; Treves et al., 2008 ▶; Ehninger and Kempermann, 2006 ▶; Taupin, 2006 ▶). The increase in DG neurons in the present study might have been induced by the effects of swimming, strategies for learning and memorizing in the MWM task, and the ischemic event. All of these factors affected all mice but only the *T. triandra-*treated groups showed a significant decrease in the percentage of dead cells in the DG region. The number of viable cells was higher than the number of dead cells. Some of the viable cells might be newborn cells from the sub-granular layer and might be stimulated by *T. triandra* leaf extract. Whether *T. triandra* leaf extract stimulates neurogenesis is left to be clarified in further studies being conducted by the authors.

Additional support for the enhancing effects of *T. triandra* leaf extract on spatial learning and learning flexibility could be found in the repetitive learning of the same cognitive task for a prolonged period of time (Ehninger and Kempermann, 2006 ▶). As described by Ragozzino (2007) ▶, the ability to shift strategies or response patterns following a change of environmental contingencies was behavioral flexibility, and it is mainly associated with brain areas such as the medial prefrontal cortex, the orbitofrontal cortex, and the dorsomedial striatum. The present study clearly revealed that *T. triandra*-treated mice showed significantly enhanced spatial learning and learning flexibility. Histological changes in these brain areas of cognitive flexibility or those brain areas of memory retrieval association, were not observed. Only the BLCCAO mice tended to spend less time in the reversal target quadrant (SW) in the reversal probe trial (the retrieval of memory of the new platform location). There might be some effect on the brain parts that support behavioral flexibility and memory. The present study only suggested that *T. triandra* leaf extract helped in acquisition process but not clearly in the retrieval process.

The present study determined that *T. triandra* leaf extract enhanced spatial learning and learning flexibility, and prevented DG neuronal death in mice with cerebral ischemia/reperfusion injury, suggesting this plant as a natural agent for prevention of neurodegenerative disease. 
